# Integrated prediction of water pollution and risk assessment of water system connectivity based on dynamic model average and model selection criteria

**DOI:** 10.1371/journal.pone.0287209

**Published:** 2023-10-19

**Authors:** Jinlou Ruan, Yang Cui, Dechen Meng, Jifeng Wang, Yuchen Song, Yawei Mao

**Affiliations:** 1 Henan Provincial Communications Planning and Design Institute Co., LTD, Zhengzhou, P.R. China; 2 Transportation Development Center of Henan Province, Zhengzhou, P.R. China; Abdul Wali Khan University Mardan, PAKISTAN

## Abstract

In recent years, with the rapid development of economy and society, river water environmental pollution incidents occur frequently, which seriously threaten the ecological health of the river and the safety of water supply. Water pollution prediction is an important basis for understanding development trends of the aquatic environment, preventing water pollution incidents and improving river water quality. However, due to the large uncertainty of hydrological, meteorological and water environment systems, it is challenging to accurately predict water environment quality using single model. In order to improve the accuracy and stability of water pollution prediction, this study proposed an integrated learning criterion that integrated dynamic model average and model selection (DMA-MS) and used this criterion to construct the integrated learning model for water pollution prediction. Finally, based on the prediction results of the integrated learning model, the connectivity risk of the connectivity project was evaluated. The results demonstrate that the integrated model based on the DMA-MS criterion effectively integrated the characteristics of a single model and could provide more accurate and stable predictions. The mean absolute percentage error (MAPE) of the integrated model was only 11.1%, which was 24.5%–45% lower than that of the single model. In addition, this study indicates that the nearest station was the most important factor affecting the performance of the prediction station, and managers should pay increased attention to the water environment of the control section that is close to their area. The results of the connectivity risk assessment indicate that although the water environment risks were not obvious, the connectivity project may still bring some risks to the crossed water system, especially in the non-flood season.

## Introduction

With the rapid development of industrialization and urbanization, the problem of water pollution caused by the concentrated discharge of domestic and industrial wastewater is becoming increasingly serious [1]. Frequent outbreaks of water pollution incidents worldwide have brought serious challenges to human safety and sustainable development. Prevention and treatment of water pollution has become an important proposition for global development. As one of the key measures to prevent and control water pollution, water quality prediction reveals changes in the river water environment in the future [2], which is of great significance for the rational development and utilization of water resources, prevention of water pollution incidents, and timely improvement of river water quality.

In recent years, many prediction methods, including statistical prediction [3], mechanism models [4] and machine learning (deep learning) [5], have been developed to simulate and predict river water quality [3, 5–7]. Among them, data-driven machine learning methods have attracted the attention of scholars [1, 5]. Machine learning algorithms can explain the complex nonlinear relationship between input variables and prediction variables [8] and have the advantages of high precision, flexible customization, and easy expansion, which have been widely used in many fields of water management [9, 10]. Wang et al. developed four machine learning models (multiple linear regression, artificial neural network (ANN), random forest (RF) and extreme gradient boosting (XGBoost)) to predict estuarine water quality. The results demonstrated that ANN, RF and XGBoost models can reliably predict estuarine water quality, and XGBoost was superior to other models in all aspects [11]. Zhong et al. used seven machine learning algorithms to build a water quality prediction model for ammonia nitrogen (AN) wastewater. The results demonstrated that the gradient boosting decision tree (GBDT) and RF models showed higher prediction performance [9]. The above literature indicate that several machine learning methods have achieved good results in water pollution prediction. However, there is no consensus on which model is optimal at present [9, 11, 12]. It is challenging to find a method suitable for different forecasting situations due to the influence of the nonstationarity of data series, uncertainty of model parameters and structure, and unknown prediction tasks [13, 14]. In addition, recent research has shown that the prediction effect of a single model is always limited, and a single model may have a large uncertainty when facing different prediction scenarios [15], which leads to a large deviation of conventional water pollution prediction methods in some prediction scenarios [16]. Therefore, it is necessary to explore more accurate and stable water pollution prediction methods to meet the requirements of water pollution prevention and control in changing environment.

Model averaging is a common method to address model uncertainty and improve prediction accuracy. It extracts information from all candidate models to provide more robust prediction [17]. In recent years, a variety of model averaging methods, including simple model averaging (SMA), weighted model averaging (WMA), frequency model averaging (FMA) and Bayesian model averaging (BMA) [18–20], have been widely used in hydrology [16], meteorology [21] and environmental research [22]. The core of the model averaging method is to estimate the optimal weight of candidate models. However, the weights of candidate models estimated by the above model averaging method are fixed. Recent research has shown that this static model averaging method shows large precision loss in local real-time prediction [17]. For example, when predicting the water quality of river stations, the best explanatory variables may show significant differences at different time nodes. Therefore, although the fixed coefficient of the explanatory variable improves the overall prediction performance of the model to a certain extent, the optimal prediction variable may change in the local real-time prediction process. This change may cause significant loss of local prediction accuracy [19, 20]. In addition, some research found that if the prediction results of all candidate models were overestimated or underestimated, the performance of the model average method would be significantly lower than that of the model selection method [16, 23]. The main reason is that the model average is a weighted average of the candidate models. When the prediction results of all the candidate models are overestimated or underestimated, the weighted average results will be lower than the candidate models with the highest accuracy. To solve the above problems, based on the machine learning method, this study attempts to propose an integrated learning criterion that combines model selection and dynamic model averaging (DMA-MS) to provide a more robust and accurate water pollution prediction, which is the main innovation of this study. Moreover, based on monitoring water quality and rainfall data, this study uses DMA-MS criteria to construct an integrated prediction model of water pollution, which is rare in data-driven water pollution prediction.

In this work, the main objective is to construct a data-driven integrated model by using the proposed DMA-MS criterion to improve the accuracy of water pollution prediction. The DMA-MS criterion identifies the best time to use the dynamic model average or model selection methods based on the error distribution characteristics of the prediction results of candidate models. On this basis, the model selection strategy based on the weighted information criterion or the dynamic model averaging method based on minimizing error is used to construct the integrated model. This method provides more stable and accurate water pollution prediction by solving the optimal weight of candidate models at each time node.

## Materials and methods

### Study area and data

The Huaihe River Basin (111°55′E-121°25′E, 30°55′N-36°36′N) is located in the middle east of China, with a drainage area of 270,000 km2. The annual average temperature of the Huaihe River basin was between 11°C and 16°C, and the annual average precipitation was approximately 920 mm. The Zhoushangyong Canal of the Huaihe River basin is located in eastern Henan Province, which uses Station S1 (Kangdian station) of the Shaying River as the diversion port. The main crossing rivers of the Canal were the Wohe River, the Baohe River and the Wangyin River, and the Canal from Shaying River to Baohe River has not been connected. Ammonia nitrogen (AN) is one of the most prominent pollutants in the Huaihe River basin [24]. Therefore, the AN concentration data from 11 water quality monitoring stations (8 in the Shaying River and 3 in the Wohe River) in the Huaihe River basin were selected to build the water pollution (AN) prediction model. Toxicological research has shown that AN can not only cause excessive growth of algae but also directly harm the health of fish by damaging the nervous system, respiratory system, liver tissue and other ways [25, 26]. Therefore, based on the establishment of the water pollution prediction model, this study assesses the connectivity risk of the Zhoushangyong Canal from the Shaying River to the Baohe River.

The water quality data (AN) and hydrometeorological data (precipitation, temperature, etc.) of the Shaying River and Wohe River from 2003 to 2021 were selected to build the water pollution prediction model for Station S1 of the Shaying River and Station W1 (Fuqiao station) of the Wohe River. The monthly water quality data of 11 monitoring stations were obtained from the Hydrological Bureau of Huaihe River Water Conservancy Commission. The water quality data were sampled in the middle of each month and brought into the laboratory for analysis on the same day or the next day. The precipitation and temperature in the hydrometeorological data were selected as the input variables for the water pollution prediction model. The monthly precipitation and temperature data from 2003 to 2021 were provided by the meteorological department of Henan Province. As the water environment quality of Station S1 and Station W1 was affected by the upstream water system, the AN of all upstream monitoring stations were determined as the input variables of the model in the process of building the water pollution prediction model. Taking Station S1 of the Shaying River as an example, the dependent variable was the future AN concentration of Station S1, and the independent variables were the AN concentrations of Stations S1 to S8, regional precipitation and temperature. The dataset contains 7920 samples, of which 5544 samples from 2003 to 2016 were selected as the training data of the candidate model, 1584 samples from 2017 to 2020 were selected as the verification data of the candidate model and the training data of the integrated model, and 792 samples from 2020 to 2021 were used as validation data of the integrated model. All data were provided by the hydrological monitoring division of Huaihe River Hydrology Bureau and the observation division of the meteorological department of Henan Province, which can be viewed and obtained in S1 Data.

### Integrated framework of water pollution prediction based on DMA-MS criteria

The proposed DMA-MS model was an integrated prediction framework that was used to improve the accuracy and stability of water pollution prediction. The framework mainly includes two key processes. The first process was the selection of candidate models and the establishment of the benchmark model for the integrated prediction model. With the rapid development of artificial intelligence and data science, a series of advanced machine learning methods have been successfully applied to research on water pollution prediction. However, the applicability of different methods may vary greatly [23]. Therefore, this study selected candidate models suitable for water pollution prediction from the perspective of method applicability and used the selected candidate models to construct water pollution prediction models. The second process was to use DMA-MS criteria to build an integrated forecasting model. This criterion judges the method of integrated model construction (including model selection and dynamic model averaging) at different stations, different seasons and different times based on the error distribution characteristics of candidate models. For the case where the model selection method was applicable, a model selection strategy based on the weighted information criterion was proposed, and then the best candidate model selected according to the strategy was used to construct the integrated prediction model of water pollution. For the case where the model averaging method was applicable, a model averaging method for dynamic adjustment of candidate model weights was proposed. This method determines the optimal weight of candidate models through repeated iterations, which aims to minimize the total error. Finally, the integrated model of water pollution prediction with adaptive adjustment of candidate models along with the changes in stations and seasons was obtained by integrating the models of all stations. In addition, the optimal weight of the integrated model was updated with the addition of new samples.

#### The selection of candidate models and establishment of benchmark models

Candidate models, as the basis for constructing integration models, are important factors affecting the performance of integration models. In recent years, machine learning methods, including ANN, support vector machine, decision tree (DT) and RF, have been widely used in research on water pollution prediction. Zhong et al. [9] used seven machine learning methods (linear regression (LR), regularized linear regression (RR), kernel ridge regression (KRR), polynomial regression (PR), k-nearest neighbor (KNN), support vector regression (SVR), GBDT and RF) to build water quality prediction models and found that RF and GBDT were better than other algorithms in fitting data. The main reason is that RF and GBDT have excellent abilities to model nonlinear relationships, deal with multicollinearity, and reduce overfitting of models [27, 28]. In addition, some recent research has found that deep learning (long short-term memory network, LSTM) demonstrated amazing performance in water quality prediction [28]. LSTM can identify the nonlinear relationship between variables and their prediction variables and transfer useful information from the past to the future. Therefore, LSTM, RF and GBDT were selected as the candidate models for the integrated framework of water pollution prediction in this study.

**Fig 1:**
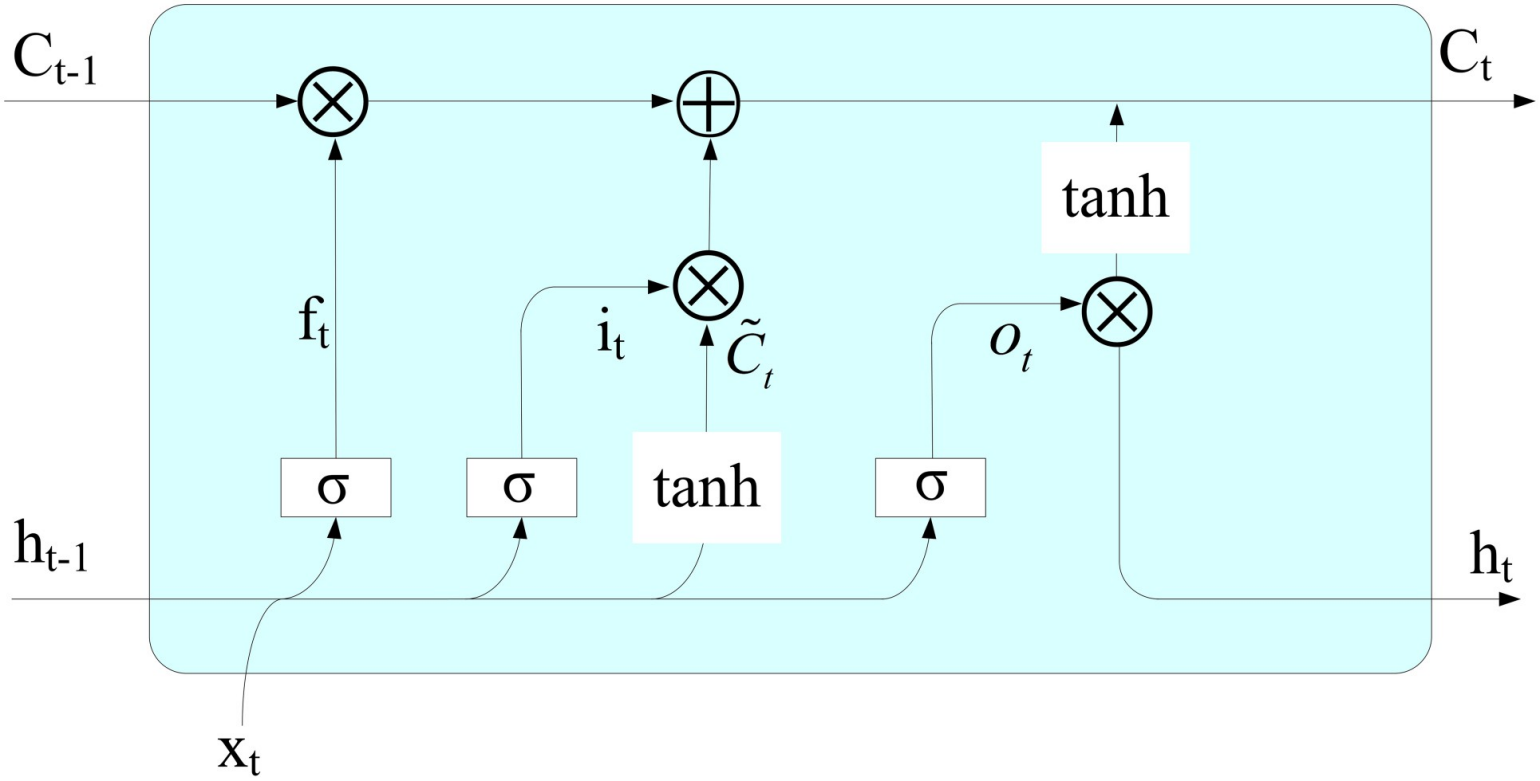
Basic principle of the LSTM algorithm.

LSTM is an improved recurrent neural network (RNN) [29]. The traditional RNN cannot closely associate the information in the previous time series with the information in the current series [30]. With the increase in the time distance of relevant information, RNN cannot maintain the long-term dependence in the input sequence [31], which often leads to the phenomenon of gradient disappearance and explosion when modeling with long time series data. To solve this problem, LSTM adds memory units to each neural unit in its hidden layer and introduces input gates, forget gates and output gates. These gating structures can store and update the cell state and achieve long-term retention of timing information [30]. As shown in Fig 1, the forget gate *f*_*t*_ controls the passing degree of the cell state *C*_*t*−1_ at time *t* − 1. The input gate *i*_*t*_ determines which information can be input into the current moment memory cell. The forget gate *f*_*t*_ and input gate *i*_*t*_ jointly update the current cell state *C*_*t*_. The output gate *o*_*t*_ determines the output of the current cell state. A detailed mathematical description of LSTM can be found in [29].

RF was a parallel integrated learning method based on DT proposed by Breiman (2001) [32], which was used to overcome the shortcomings of overfitting and instability when using the DT model. RF uses bootstrap resampling technology to repeatedly and randomly select samples from the original training sample set to train the decision tree [33]. The RF model was formed by combining all decision trees generated after training. RF randomly extracts some samples and features from the sample set each time, making it have good noise resistance and strong robustness. Although the RF model has a simple structure, it is amazing that it shows strong performance in many real tasks [34]. RF can handle both classification and regression problems. In this study, RF regression was used to construct the prediction model for water pollution (AN concentration). The main super parameters were mtry (the number of variables at each node of the tree) and ntree (the number of trees) [27].

GBDT is also an integrated learning algorithm based on DT [35]. The core of the algorithm is that each iteration fits the residuals of the DT of the previous iteration. In this study, the classification and regression tree (CART) was used as the basic learner of GBDT. In the process of modeling with GBDT, the initial regression tree was obtained by training the original training set. Then, the square loss function was used to calculate the residual of the initial regression tree, and the gradient descent algorithm was used to fit the residual of the initial regression tree to form a new decision tree. The regression tree with gradually reduced residuals was generated through repeated iterations. When the residual error of the regression tree was lower than the allowable error, the iteration was stopped, and the GBDT model was obtained by accumulating the regression trees of each iteration [36]. The learning rate (*ε*) and complexity (*C*) were the parameters that had the most obvious impact on the precision of the GBDT model [37]. *C* represents the depth of the tree of the GBDT model. The greater *C* is, the higher the complexity of the model. *ε* indicates the weight reduction coefficient of each regression tree (the value of *ε* is greater than 0 and less than 1); the smaller is, the *ε* better the fitting effect of the model, but correspondingly more trees are required to fit the model, resulting in an increase in the number of iterations. In general, the model will generally achieve better results when *ε* is set to 0.001. Therefore, *ε* was set to 0.001 in this study. C was set to 0.1 and 0.001 for parameter optimization.

LSTM, RF and GBDT were used to construct the water pollution prediction model (AN concentration) for each station. To improve the performance of the candidate models, the parameters of LSTM, RF and GBDT were optimized using the Monte Carlo method. Monte Carlo was a nonparametric estimation method that uses a series of sampling points to approximate the probability distribution. The core of the Monte Carlo method was mass sampling and gradual approximation. The more simulations there are, the better the effect of parameter estimation, but the running time of the model will be increased. Considering the effect of parameter optimization and calculation efficiency, the number of Monte Carlo samples was set to 10000 in this study.

#### DMA-MS criterion

Based on the prediction results of the three candidate models, an integrated prediction criterion was proposed. The criterion includes three key processes: the identification of the integrated prediction method, the model selection strategy based on the weighted information criterion, and the dynamic model averaging based on error minimization. Due to the difference in upstream water system characteristics and the number and location of monitoring stations, the input variables of models at each station may be quite different. Therefore, an independent integrated prediction model for each station was established. In addition, recent research has shown that the runoff and infiltration laws in the flood season and non-flood season are quite different [38]. The precipitation and runoff in the flood season were larger than those in the non-flood season. Under a similar pollutant discharge, the larger runoff will improve the pollutant carrying capacity of the water body. However, greater precipitation will increase the dissolution and infiltration of pollutants, leading to an increase in the total amount of pollutants in the water body. Therefore, to avoid the impact of the difference in pollutant concentration in flood and non-flood seasons on the model performance, an independent integrated prediction model for flood season and non-flood season was established.

(1) The identification of the integrated prediction method

The identification of an integrated prediction method refers to using model selection or model comments to construct the integrated prediction model by analyzing the prediction results of the candidate models. Assuming that the errors of the candidate models follow the normal distribution (Eq 1), if the prediction results of each candidate model were overestimated or underestimated, the model selection strategy based on the weighted information criterion was used to construct the integrated prediction model of water pollution. In contrast, the dynamic model average method based on error minimization was used to construct the integrated prediction model.

$$y_{t}^{k}∼N({\bar{y}}_{t}^{k},\sigma_{t}^{k})$$ (1)

$$f_{t}=\left\{\begin{array}{cc} MA & \forall ({\bar{y}}_{t}^{k}-y_{t}^{k})>0∥\forall ({\bar{y}}_{t}^{k}-y_{t}^{k})<0\\ MS & esle \end{array}\right.$$ (2)

where $y_{t}^{k}$ is the predicted value of the k-th candidate model at time t, ${\bar{y}}_{t}^{k}$ is the average of the predicted value of the k-th candidate model at time t, and $\sigma_{t}^{k}$ is the variance of the predicted value of the k-th model at time t.

(2) The model selection strategy based on the weighted information criterion

The model selection strategy based on the weighted information criterion (WIC) can comprehensively reflect the performance of each candidate model [39]. Considering the demand for model accuracy in water pollution prediction, the mean absolute percentage error (MAPE), root mean square error (RMSE) and DA [40, 41] were selected as the main basis for the model selection strategy. MAPE and RMSE reflected the prediction accuracy of the candidate models. DA reflected the forecast trend and accuracy. Finally, the candidate model with the smallest WIC was selected to establish the integrated prediction model of water pollution. This model selection strategy comprehensively reflects the performance of each candidate model in terms of model accuracy and prediction trend. It should be noted that the complexity of the candidate model may also be an important factor to consider in the selection of candidate models. However, the time scale of water pollution prediction is relatively large in this study (monthly), and the complexity of the candidate model will hardly affect the timeliness of prediction models. Therefore, model complexity was not considered in this study.

$$MAPE=\frac{1}{n}\sum_{\text{i}=1}^{n}\left|\frac{y_{i}-{\hat{y}}_{i}}{y_{i}}\right|\times 100\text{\%}$$ (3)

$$RMSE=\sqrt{\frac{\sum_{\text{i}=1}^{n}(y_{i}-{\hat{\text{y}}}_{i}{)}^{2}}{n}}$$ (4)

$$DA=\frac{1}{n-1}\sum_{i=1}^{n-1}a_{i},\;\;\;a_{i}=\left\{\begin{array}{cc} 1 & if(y_{i+1}-{\hat{y}}_{i})({\hat{y}}_{i+1}-y_{i})>0\\ 0 & otherwise \end{array}\right.$$ (5)

WIC=0.5(MAPE+RMSE)+0.5DA (6)

where *y*_*t*_ is the measured value, ${\hat{y}}_{t}$ is the predicted value of the sample, and n is the total number of samples for integrated prediction model construction.

(3) Dynamic model averaging based on error minimization

The dynamic model averaging method can integrate the results of multiple candidate models to provide more robust prediction. The main idea of using the dynamic model average based on error minimization to predict water pollution was to give the best weight based on the prediction characteristics of candidate models. First, the initial weight of candidate models was calculated according to the sum of squared error (SSE) in the prediction results of each candidate model (Eq 8). Then, with the goal of reducing the total error, the weight of each candidate model was adjusted iteratively until the deviation was lower than the allowable error. The specific calculation processes were as follows:

Step 1. Initialize the weight of candidate models *ω*(0).
$$\omega (0)=[\omega_{1}^{},\omega_{2}^{},\ldots ,\omega_{k}^{}]$$ (7)
ωk=1SSEk1∑i=1kSSEi (8)
  where *ω*_*k*_ is the weight of the k-th candidate model, *SSE*_*k*_ is the sum of squares due to the error of the k-th candidate model, and k is the number of candidate models.
Step 2. Calculating the initial result ${\hat{y}}_{t}(0)$ and deviation *ε*_*t*_(0) of the integrated prediction model at time t.
$${\hat{y}}_{t}(0)=\omega (0){\hat{y}}_{t}^{i},i=1,2,\ldots ,k$$ (9)
$$\varepsilon_{t}(0)={\hat{y}}_{t}(0)-y_{t}$$ (10)
  where ${\hat{y}}_{t}^{i}$ is the predicted value of the i-th candidate model at time t. *y*_*t*_ is the measured value at time t.
Step 3. Adjust the weight of the candidate models *ω*_*t*_(1) according to the residual of the initial prediction results, repeat this step m times, stop iteration when the prediction error meets the requirements, and obtain the best weight *ω*_*t*_(*m*) of the candidate model.
$$\omega_{t}(1)=\left\{\begin{array}{cc} \omega (0)-\frac{{\left({\hat{y}}_{t}(0)-y_{t}\right)}^{2}}{\lambda \sum_{i=1}^{k}SSE_{i}} & if\;({\hat{y}}_{t}(0)-y_{t})>0\\ \omega (0)+\frac{{\left({\hat{y}}_{t}(0)-y_{t}\right)}^{2}}{\lambda \sum_{i=1}^{k}SSE_{i}} & if\;({\hat{y}}_{t}(0)-y_{t})<0 \end{array}\right.$$ (11)
$$\omega_{t}(m)=[\omega_{1}^{}(m),\omega_{2}^{}(m),\ldots ,\omega_{k}^{}(m)]$$ (12)
  where *λ* is the weight adjustment coefficient, and the value of *λ* is an integer greater than or equal to 1. The higher *λ* is, the higher the number of iterations.

In the process of water pollution prediction, with the addition of new samples, the optimal weights of the candidate models are adjusted by repeating the above steps to achieve dynamic updates of the weights of the candidate models.

### Risk assessment of connectivity projects based on water pollution prediction

Based on the prediction results of the integrated water pollution model, the risk quotient model [26, 42, 43] was used to assess the water environmental risks of Station S1 in the Shaying River and Station W1 in the Wohe River, and the weighted average value of the water ecological risk at Stations S1 and W1 was used to characterize the connectivity risk of the connectivity project from the Shaying River to the Wohe River. As shown in Eq 13, the risk quotient of the station mainly depends on the AN concentration of the station (EEC) and the standard value (WQC). The standard value of AN was divided into short-term standards and long-term standards. Since the monthly AN concentration data was selected as the sample data, the long-term standard (Eq 14) was selected to calculate the standard value of AN. The risk grade was determined according to the risk quotient value obtained. When RQ<1, it was considered that there was basically no risk; 1 ≤ RQ < 10 was risky; RQ ≥ 10 was high risk [44, 45].

$$RQ\text{=}\frac{EEC}{WQC}$$ (13)

$$WQC=(\frac{0.0339}{1+10^{7.688-pH}}+\frac{1.46}{1+10^{pH-7.688}})\times MIN(2.852,0.914\times 10^{0.028\times (25-MAX(t,7))})$$ (14)

where *EEC* is the environmental exposure concentration, which refers to the concentration of AN (mg/L) at the station in this study; *WQC* is the water quality criteria (mg/L), which refers to the AN criteria value related to aquatic organisms; *pH* is the hydrogen ion concentration; and *t* is the water temperature.

## Results and discussion

### Prediction performance analysis of candidate models

**Fig 2:**
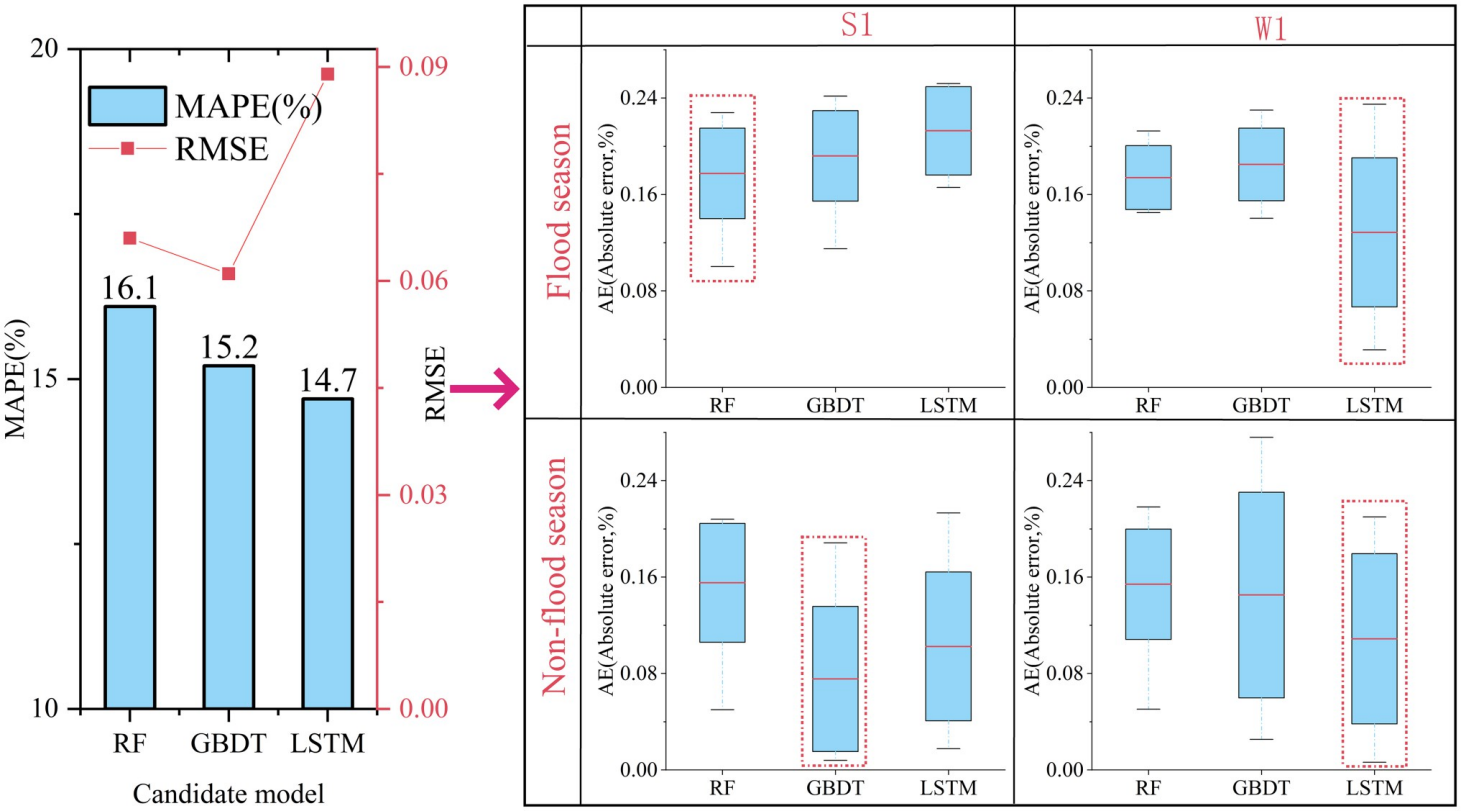
Overall precision (left) and local precision (right) of the prediction results of the candidate models.

The AN, precipitation and temperature data of 11 stations in the Huaihe River basin from 2003 to 2016 were used to construct the candidate models (RF, GBDT and LSTM) for water pollution prediction. Before model construction, the 3σ principle was used to analyze and eliminate the abnormal data in the input data. In addition, to eliminating the impact of data dimension and singular sample data on model prediction performance, the maximum and minimum normalization method [46, 47] was used to normalize the input data. In the process of model construction, the sample data of 2016 was selected to calibrate the parameters of candidate models. To verify the prediction performance of the candidate models, the test dataset from 2017 to 2020 was used for model evaluation. As shown in Fig 2, the MAPE of the predicted results of the three candidate models was between 14.7% and 16.1% (on the left side of Fig 2), indicating that each candidate model can better predict the water pollution status of the monitoring stations. Among the three candidate models, LSTM had the lowest MAPE, showing the highest prediction performance. However, the right side of Fig 2 shows that the fluctuation degree of the prediction error of the LSTM model was significantly higher than that of the RF and GBDT models, which indicates that although the overall accuracy of LSTM was higher, the RMSE of the model was significantly higher than that of the RF and GBDT models.

In addition, the right side of Fig 2 also reflects the prediction performance differences of candidate models at different stations and in different seasons. The prediction performance of the three candidate models in the non-flood season was obviously better than that in the flood season. The main reason was that the precipitation and precipitation intensity in the flood season were both large. The rainwater entered the river through the process of runoff generation, confluence and infiltration after the rainwater reached the ground. During this process, rainwater dissolves a large amount of pollutants flowing into the river [48, 49], leading to greater uncertainty in the water environment quality of the river water body [7, 50]. It is worth noting that the candidate model performance of each station was quite different. At Station S1, the RF model has a low prediction error in the flood season, while GBDT has a low prediction error in the non-flood season. At Station W1, the LSTM model always has the lowest prediction error. These results demonstrated that the performance of a single model in different prediction tasks was always limited. Therefore, based on the prediction characteristics of candidate models, an integrated prediction model for water pollution was constructed in this study, which aims to provide more robust prediction.

### The results and prediction performance of the integrated model

**Fig 3:**
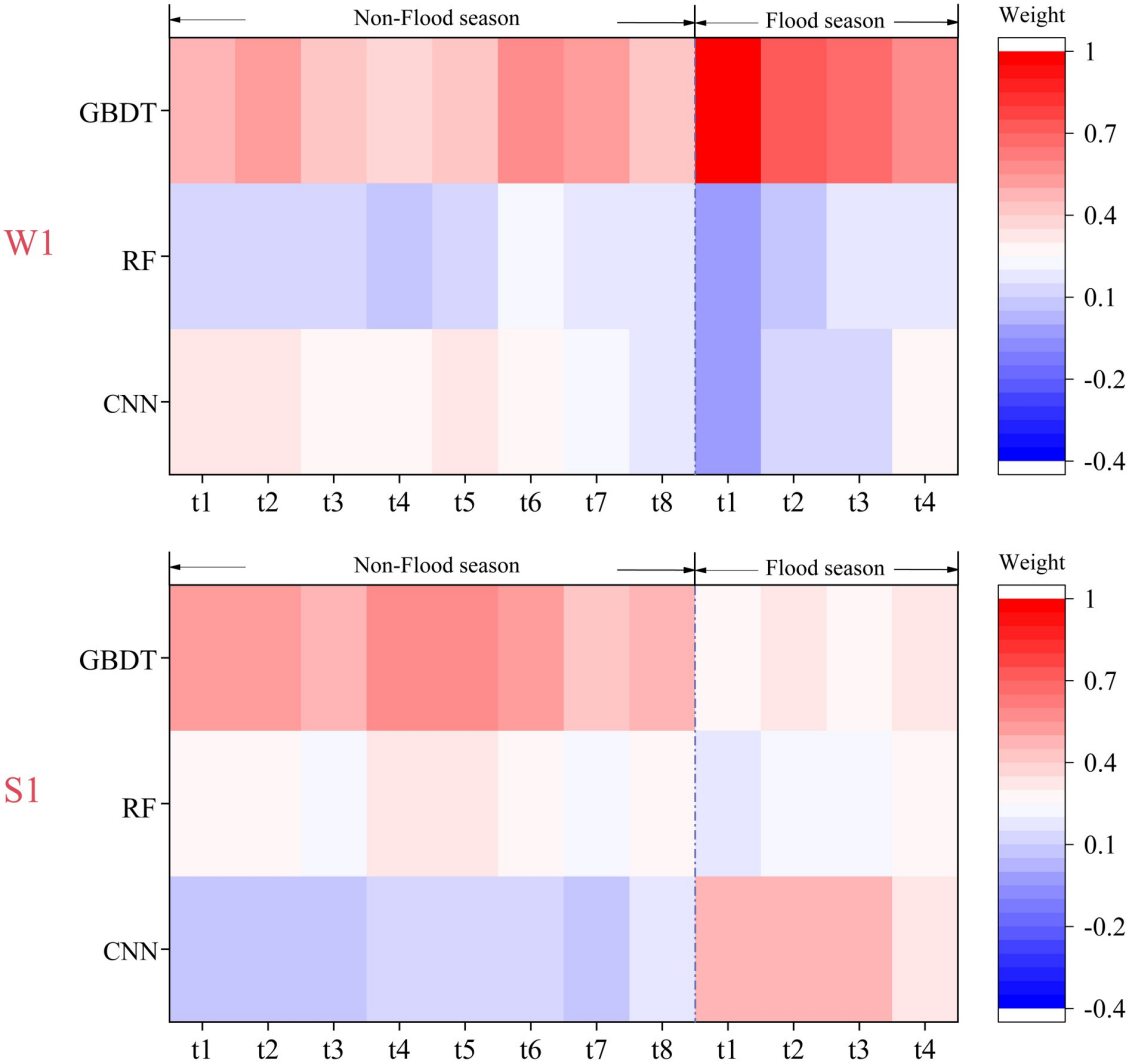
Weights of candidate models in the integration model.

The prediction results of the candidate models in 2017–2020 were selected as the training samples of the integrated model, and the DMA-MS method was used to determine the optimal weight of the candidate models to construct the integrated prediction model for each station and different seasons. Sample data from January to December 2021 were used to test the prediction performance of the integrated model. Fig 3 shows the optimal weight of candidate models for each station and different seasons in the integrated model. LSTM has a higher weight at Station W1 because the LSTM model has a low prediction error, which is consistent with the results in Fig 3. At Station S1, the RF model has the highest weight in the prediction of the flood season, and GBDT has a higher weight in the prediction of the non-flood season. This result also showed good consistency with the prediction performance of candidate models (Fig 3), indicating that the performance of candidate models was an important factor determining their weight in the integrated model. In addition, at time t1 in the flood season prediction of Station W1, the optimal weight of GBDT was 1, and the optimal weight of the LSTM and RF models was 0, indicating that the GBDT model was determined to be the integrated prediction model at this time. The reason is that the prediction results of all candidate models at this time were overestimated or underestimated, and the model average method can no longer improve the accuracy of the prediction results. Therefore, according to the identification criterion of the integrated prediction method, the candidate model (GBDT) with the highest prediction performance was selected as the integrated prediction method. This result indicates that it is necessary to establish an integrated model based on model selection and model averaging.

**Fig 4:**
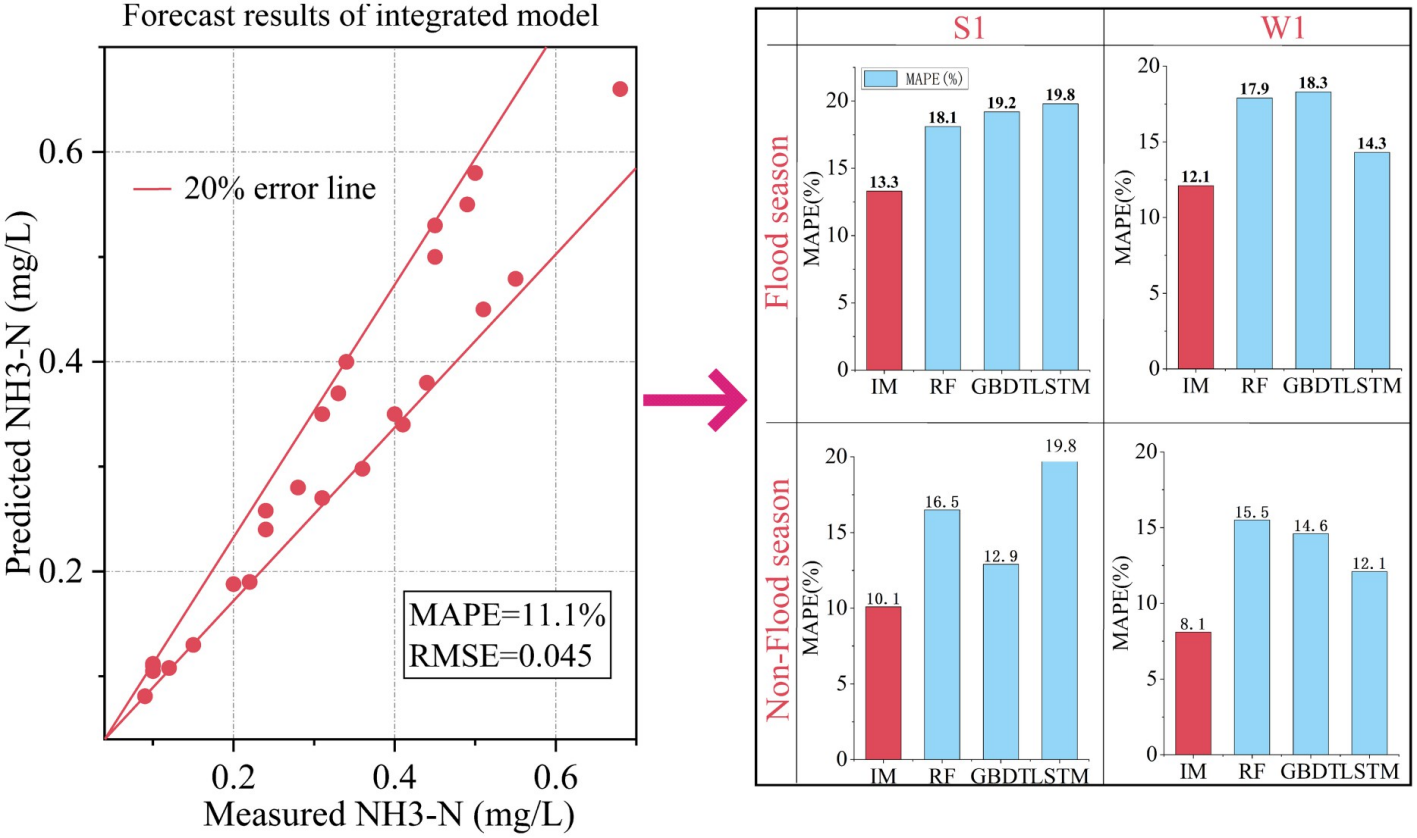
Performance of the integrated model (IM refers to the integrated model).

Fig 4 shows the prediction results and performance of the integrated models. The MAPE of the integrated model (11.1%) was 24.5%–45.0% lower than that of the candidate models (RF, GBDT and LSTM). The RMSE (0.045) of the integrated model was also significantly lower than that of the candidate models. In addition, the integrated model always has low prediction error at different monitoring stations and seasons, indicating that the integrated prediction model of water pollution based on the DMA-MS criterion can effectively integrate the advantages of a single model to improve the accuracy of the prediction results. The prediction accuracy of the integrated model in the non-flood season (with MAPEs of 10.1% and 8.1%) was significantly better than that in the flood season (with MAPEs of 13.3% and 12.1%). The main reason was that the water quality in the flood season was uncertain due to the influence of rainfall, and the candidate model had a larger error in the prediction of the flood season, leading to the prediction performance of the integrated model in the flood season being obviously inferior to that in the non-flood season. Nevertheless, the proposed integrated model shows outstanding advantages in improving the prediction accuracy of a single model.

Water pollution prediction has always been a hot research topic for scholars. Zamri et al. compared the performance of 10 machine learning algorithms in pollution source classification, and found that RF algorithm has high performance in pollution source classification prediction (with 98.78% accuracy) [51]. The main reason was that RF can integrate multiple decision tree models to provide more stable prediction. These results also indicated that it is reasonable to select RF and GBDT as candidate models in this study. In addition, several recent researches have confirmed that the integrated model can significantly improve the prediction performance of water pollution [10, 52]. However, there are certain differences between previous researches and the DMA-MS model proposed in this study in terms of prediction performance. For example, Li et al. proposed a new mixed model to predict the AN of surface water [52]. The RMSE of the test results was 0.054–0.286, which was more than 15% higher than the DMA-MS model. Therefore, compared with previous researches, the integrated prediction method of water pollution prediction proposed in this study may has better prediction accuracy and stability, which may provide reference for improving the water pollution prediction method.

### Feature importance analysis

**Fig 5:**
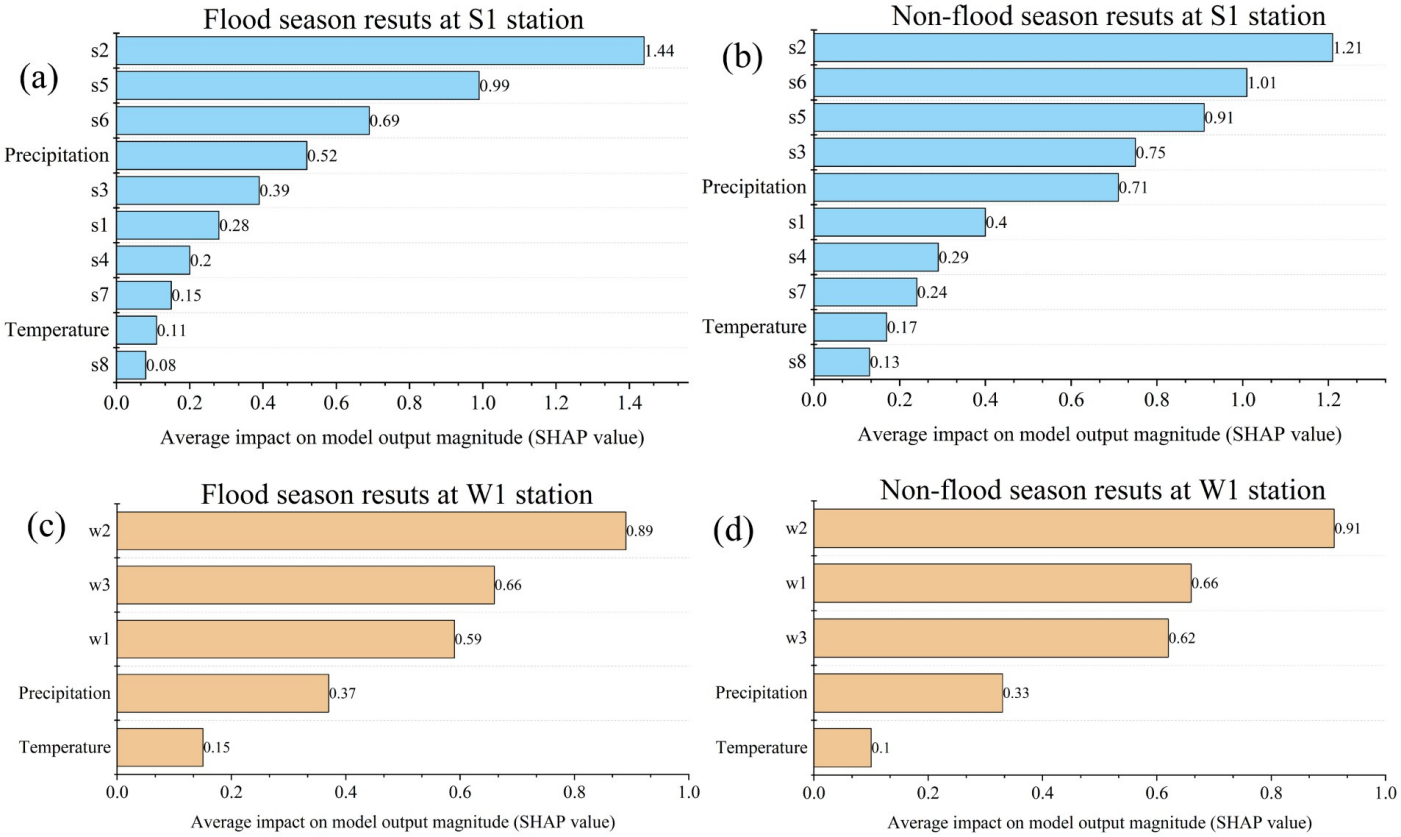
Results of feature importance analysis.

Feature importance analysis can reflect the impact of input variables on model output, clarify the source and mechanism of water pollution, and provide a reference for water pollution treatment. The Shapely additive explanations (SHAP) method [21], which can display the influence degree of each sample feature, was used to calculate the influence degree of input variables (water quality of each station, precipitation and temperature) on the output [11]. The average SHAP value of the input variable reflects the impact of the input variable on the model output. The larger the average SHAP value is, the greater the impact on water pollution prediction. As shown in Fig 5, regardless of the flood season or non-flood season, Stations S2, S5 and S6 have a great impact on the output of the model in the prediction of water pollution for Station S1. Among them, Station S2 has the greatest impact on the output of the model, which indicates that the nearest station has a greater impact on the output of the model. In addition, although the influence of precipitation on model output was lower than that of Stations S2, S5 and S6, it was also an important factor affecting model output. In the prediction of water pollution for Station W1, Stations W2, W3 and W1 have a great impact on the model output, and Station W2 has the greatest impact on the model output. Therefore, these results demonstrate that in the water pollution prediction of different stations, the stations closer to the predicted station have a greater impact on the water pollution prediction, which was consistent with the research of [11]. Therefore, in the process of water pollution control and management, managers should focus on and adjust the water environment of the control section that is closest to their area. These results can provide a reference for regional water environment protection and governance.

### Analysis of the water environment risk of the connection project

**Fig 6:**
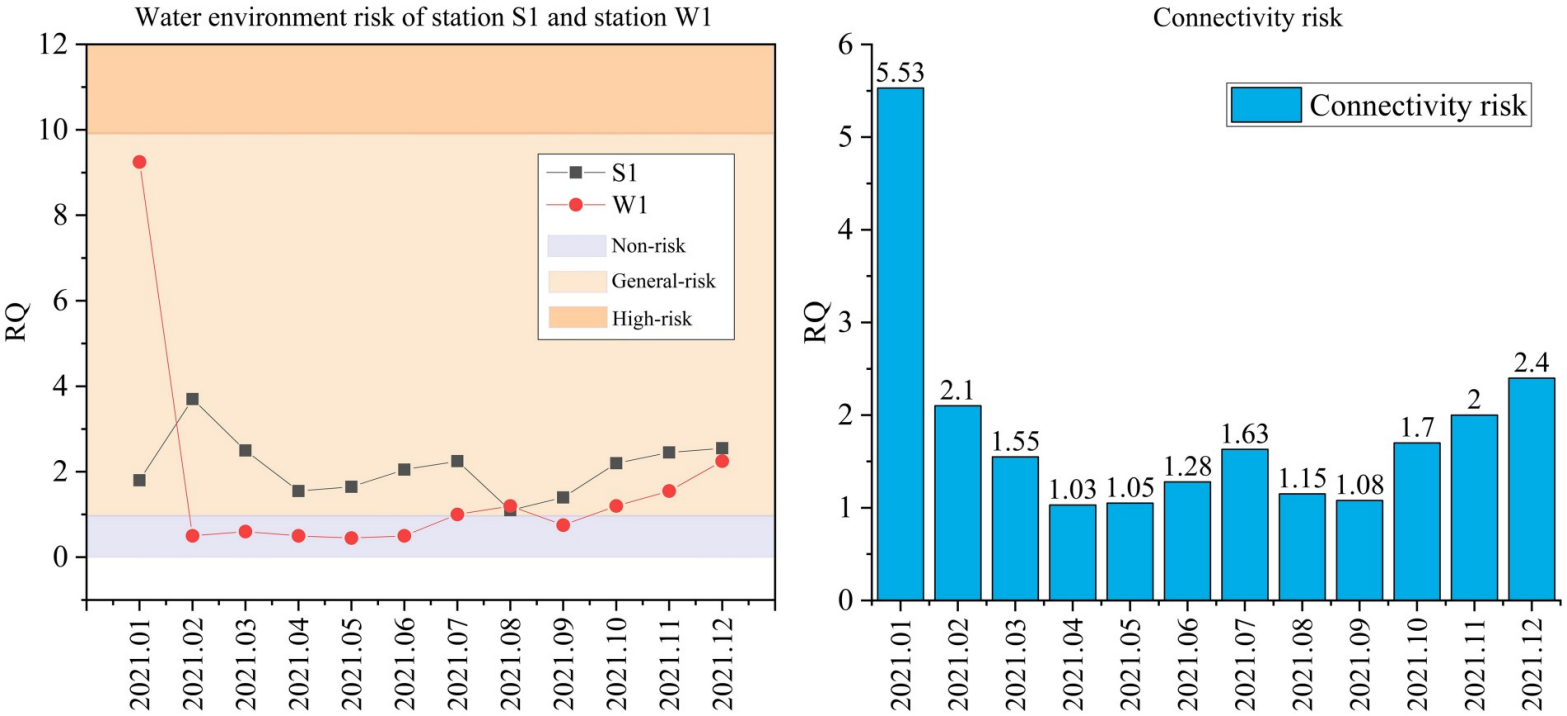
Risk analysis results of the Zhoushangyong canal connection project.

Based on the water pollution prediction results of the integrated model from January to December 2021, the risk quotient model was used to assess the connectivity risk of the Zhoushangyong Canal from the Shaying River to the Baohe River. As shown in Fig 6 (left), the water environmental risk of Station S1 was low throughout the year. The water environment risk of Station W1 was high in January but low in other months. In addition, the water environment risk of Station W1 was obviously lower than that of Station S1 from February to December. Because the water of the Shaying River was diverted from Station W1 to the connectivity project, the water environment of Station S1 of the Shaying River may bring some risks to the Wohe River. Fig 6 (right) reflects the connection risks of the connection project at different times. Although the RQ value of the connection risk of the Zhoushangyong Canal from the Shaying River to the Baohe River was lower than 10, the connection risk in the non-flood season was obviously higher than that in the flood season, and the time of higher connection risk was mainly from October to February. The main reason was that the autumn and winter seasons were the golden period of China’s industrial production, and the industrial pollutant emissions were relatively large, but the precipitation in the Huaihe River basin was relatively small in the same period, resulting in the water environment risk of the connectivity project being higher in autumn and winter. Therefore, to reduce the water environmental risk of the connectivity project, it is recommended that the connectivity project be constructed from March to September.

## Conclusion

Based on the proposed DMA-MS criterion, this study constructed an integrated prediction framework for water pollution that integrated model selection and dynamic model averaging. First, based on the selection and construction of candidate models, an integrated model identification mechanism based on the prediction features of candidate models was proposed, which could effectively identify the best time to use the dynamic model average or model selection methods. On this basis, the integrated prediction model of water pollution was constructed by using the model selection strategy based on the weighted information criterion and dynamic model averaging based on error minimization. The main conclusions are as follows:

1. The prediction performance of each candidate model at different stations and in different seasons was quite different, indicating that the prediction performance of a single model was limited. At Station W1, LSTM always has a better prediction performance, while at Station S1, RF has a higher prediction accuracy in the flood season, and GBDT has a higher prediction accuracy in the non-flood season.
2. The integrated model based on DMA-MS effectively integrated the characteristics of a single model and could provide more accurate and stable prediction. The MAPE of the integrated model was only 11.1%, which was 24.5%–45% lower than that of the single model.
3. The results of the analysis demonstrate that the nearest station was the most important factor affecting the performance of the prediction station. As such, in the process of water pollution control and management, managers should focus on and adjust the water environment of the control section that is closest to their area.
4. Although the water environment risks of Station S1 in the Shaying River and Station W1 in the Wohe River were not obvious, the connectivity project will still bring some risks to the crossed water system. To reduce the water environmental risk of the connectivity project, it is recommended that the connectivity project be constructed from March to September.

However, due to the limitation of sample data, this study only considered the impact of water quality, precipitation and temperature on water pollution prediction. Future research can explore factors affecting water pollution prediction to improve performance. Nevertheless, this study proposes an integrated learning framework that integrates model averaging and model selection, with great potential for scalability. On the one hand, this method can be used not only to integrate different machine learning algorithms but also to integrate numerical models with physical mechanisms. On the other hand, this method can not only be used to provide more robust water pollution prediction but also be used in runoff prediction, precipitation prediction and other research.

## Supporting information

S1 Data (XLSX)
